# Investigation of Optimum Mg Doping Content and Annealing Parameters of Cu_2_Mg*_x_*Zn_1−*x*_SnS_4_ Thin Films for Solar Cells

**DOI:** 10.3390/nano9070955

**Published:** 2019-06-30

**Authors:** Yingrui Sui, Yu Zhang, Dongyue Jiang, Wenjie He, Zhanwu Wang, Fengyou Wang, Bin Yao, Lili Yang

**Affiliations:** 1 Key Laboratory of Functional Materials Physics and Chemistry of the Ministry of Education, Jilin Normal University, Siping 136000, China; 2 State Key Laboratory of Superhard Materials and College of Physics, Jilin University, Changchun 130012, China

**Keywords:** Cu_2_Mg*_x_*Zn_1−*x*_SnS_4_, thin films, photoelectric performance, sol–gel, sulfuration treatment, solar cell

## Abstract

Cu_2_Mg*_x_*Zn_1−*x*_SnS_4_ (0 ≤ *x* ≤0.6) thin films were prepared by a simple, low-temperature (300 °C) and low-cost sol–gel spin coating method followed by post-annealing at optimum conditions. We optimized the annealing conditions and investigated the effect of Mg content on the crystalline quality, electrical and optical performances of the Cu_2_Mg*_x_*Zn_1−*x*_SnS_4_ thin films. It was found that the Cu_2_Mg*_x_*Zn_1−*x*_SnS_4_ film annealed at 580 °C for 60 min contained large grain, less grain boundaries and high carrier concentration. Pure phase kesterite Cu_2_Mg_*x*_Zn_1−*x*_SnS_4_ (0 ≤ *x* ≤ 0.6) thin films were obtained by using optimal annealing conditions; notably, the smaller Zn^2+^ ions in the Cu_2_ZnSnS_4_ lattice were replaced by larger Mg^2+^ ions. With an increase in *x* from 0 to 0.6, the band gap energy of the films decreased from 1.43 to 1.29 eV. When the ratio of Mg/Mg + Zn is 0.2 (*x* = 0.2), the grain size of Cu_2_Mg*_x_*Zn_1−*x*_SnS_4_ reaches a maximum value of 1.5 μm and the surface morphology is smooth and dense. Simultaneously, the electrical performance of Cu_2_Mg*_x_*Zn_1−*x*_SnS_4_ thin film is optimized at *x* = 0.2, the carrier concentration reaches a maximum value of 3.29 × 10^18^ cm^−3^.

## 1. Introduction

In recent years, the semiconductor Cu_2_ZnSnS_4_ (CZTS) has attracted enormous attention as an ideal absorber material for low-cost thin film solar cells. For thin film CuInGaSe_2_ (CIGS) and CdTe solar cells, reliable efficiencies of more than 20% have been achieved [1,2]. However, the limited resources and extremely high costs of In and Ga, and toxicity of Se and Cd significantly limit further development of CIGS and CdTe solar cells. CZTS is regarded as a substitute for CIGS, wherein the high-cost and rare In and Ga, and toxic Se are replaced by low-cost and earth-abundant Zn, Sn and S, respectively. In addition to being composed of abundant and non-toxic elements, CZTS exhibits remarkable photoelectric properties as an absorbing layer, including a high absorption coefficient (>10^4^ cm^−1^) and a suitable band gap (1.40–1.50 eV) [3]. To date the best efficiency of pure CZTS has broken through 11% [4,5], but it is still far below than that of CIGS (21.7%) [6]. In order to realize the industrialization of low-cost and environmental protection CZTS solar cells, it is necessary to further improve the efficiency of CZTS based thin film solar cells. The low efficiencies of CZTS solar cells are attributed to factors such as low crystallinity, large open circuit voltage (V_oc_) deficit as well as poor band alignment at the CdS/CZTS interface [7,8,9]. As we all know, V_oc_ is linearly related to the band gap of CZTS. Band gap engineering has emerged as an effective method to adjust the band alignment at the CdS/CZTS heterojunction interface [10,11]. Therefore, it is extremely crucial to find the absorption layer that is conducive to tuning the band gap of CZTS.

The band gap of CZTS can be decreased from 1.5 eV to 1.0 eV by doping Se and tuning the S/Se ratio [12], which is a widely used method to tune the band gap of CZTS. However, it is not easy to precisely control the S/Se ratio during the annealing process, because of the difference in the volatility of S and Se [13,14,15]. In addition, Se is extremely hazardous to human health and the environment. Cation substitution is considered a highly effective means to tune the band gap of CZTS, particularly the substitution of Zn with Cd, the latest efficiency has exceeded 11% [5]. Several studies have demonstrated that via Cd doping, the band gap of CZTS can be adjusted and the crystalline quality of the CZTS films can be effectively improved, leading to a significant improvement in the V_oc_ of CZTS [16,17]. However, Cd toxicity is a serious issue. In addition, several other elements have been introduced to CZTS, such as Sb and alkali metals (Na, K) [18,19,20,21,22,23]. However, it is found that these doping elements have little influence on the tuning band gap, because the ionic radii of these elements are mismatched, leading their incorporation only on the grain boundaries or surfaces rather than in the CZTS lattice. Therefore, the development of a facile and environmentally friendly method to tune the band gap of CZTS is highly imperative.

Tuning the band gap of CZTS by substituting Zn with Mg is more advantageous compared to that with other elements. Firstly, compared to Na, K and Sb ions, Mg^2+^ ions occupy Zn^2+^ sites in the CZTS lattice rather than segregate on the CZTS grain boundaries and surfaces, because the radii of Mg^2+^ and Zn^2+^ are very similar. In addition, the introduction of Mg in the CZTS absorber layer has unique strengths because of the low price, high reserves and being environmentally friendly. Mg is more abundant than Zn, less costly than Ge and environmentally friendly compared to Cd [24]. Lastly, some possible impurity phases due to the existence of ZnS during the synthesis process of the CZTS precursor solution may be eliminated or reduced. Since the ZnS binary phase exists stably in the solution condition but the MgS binary phase is unstable [24]. These advantages make the substitution of Zn with Mg an effective approach to adjust the band gap of the CZTS. So far, the research about Cu_2_MgSnS_4_ nanoparticles prepared by hot-injection have been reported [24], in our previous studies, the Cu_2_Mg*_x_*Zn*_x_*Sn(S,Se)_4_ thin films with different Mg concentration have been successfully synthesized by the sol–gel method [25], but the study of Cu_2_Mg*_x_*Zn_1−*x*_SnS_4_ thin films as the absorption layer has not been investigated.

In this work, it is the first time Cu_2_Mg*_x_*Zn_1−*x*_SnS_4_ thin films with different Mg content were fabricated by the sol–gel method. The crystal structure and electro-optic performance of the Cu_2_Mg*_x_*Zn_1−*x*_SnS_4_ films were systematically characterized. It is found that Mg^2+^ ions were successfully incorporated into the CZTS lattice, which occupied the Zn^2+^ ion sites, the prepared Cu_2_Mg*_x_*Zn_1−*x*_SnS_4_ thin films had kesterite structures, indicating that Mg doping did not affect the crystal structure. Moreover, Mg doping resulted in an increase in the particle size and enhancement in the crystallinity and electrical properties of the CZTS film. With an increase in *x* from 0 to 0.6, the band gap of the Cu_2_Mg*_x_*Zn_1−*x*_SnS_4_ film decreased from 1.43 to 1.29 eV.

## 2. Experimental Method

### 2.1. Sample Preparation

Soda lime glass (SLG) substrates were used to deposit Cu_2_Mg*_x_*Zn_1−*x*_SnS_4_ thin films. Firstly, Cu_2_Mg*_x_*Zn_1−*x*_SnS_4_ precursor solutions with different Mg contents were prepared by the sol-gel method. The copper (II) acetate monohydrate (0.8086 g), tin (II) chloride hydrate (0.5077 g), zinc acetate and magnesium chloride hexahydrate (0.4794 g) were dissolved in 2-methoxyethanol (10 mL) and stirred for 10 min, then the solution was evenly mixed with thiourea (1.3702 g). For the sake of obtaining precursor solutions with different Mg contents, the mole ratios of Mg/(Mg + Zn) were set to 0, 0.1, 0.2, 0.4 and 0.6 in the solution. After the complete dissolution of the metal compounds, monoethanolamine was added as a stabilizer, and the stirring process was continued until the solution became clear and transparent.

Cu_2_Mg*_x_*Zn_1−*x*_SnS_4_ thin films were deposited onto SLG substrates by spin coating at 3000 rpm for 30 s, followed by sintering for 5 min on a hot plate at 300 °C in air. To obtain micrometer thick Cu_2_Mg*_x_*Zn_1−*x*_SnS_4_ films, the coating and sintering processes were repeated. Next, the precursor films were rapidly annealed under a sulfur atmosphere at different annealing temperatures and times.

### 2.2. Materials Characterization

The crystal structures of the prepared Cu_2_Mg*_x_*Zn_1−*x*_SnS_4_ thin films were characterized by power X-ray diffraction (XRD; λ = 0.15406 nm/max-ga, Rigaku Corporation, Tokyo, Japan) and Raman spectroscopy with an excitation wavelength of 514 nm. The chemical composition of the Cu_2_Mg*_x_*Zn_1−*x*_SnS_4_ films and the chemical bonding states of the constituents were characterized by X-ray photoelectron spectroscopy (XPS; Thermo Fisher Scientific, Waltham, MA, USA) using Al Kα as the X-ray source. Scanning electron microscopy (SEM) was performed using Hitachi S-4800 (JEOL Ltd., Tokyo, Japan). The electrical properties of the Cu_2_Mg*_x_*Zn_1−*x*_SnS_4_ films were measured in the van der Pauw configuration. The optical properties of the films were measured using an ultraviolet-visible-near-infrared (UV-Vis-NIR) spectrophotometer (UV-3101PC, Tokyo, Japan).

## 3. Results and Discussion

**Figure 1:**
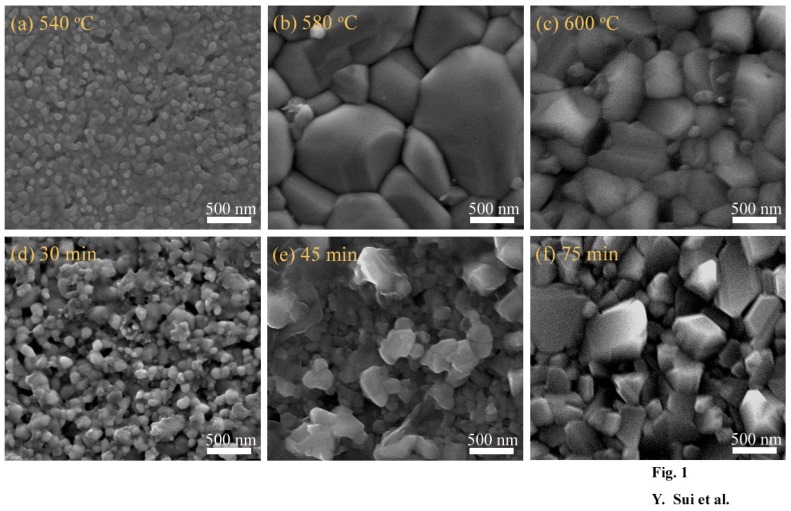
Scanning electron microscopy (SEM) images of Cu_2_Mg*_x_*Zn_1−*x*_SnS_4_ (0 ≤ *x* ≤ 0.6) thin films annealed at a different annealing condition: (**a**) 540 °C, 60 min; (**b**) 580 °C, 60 min; (**c**) 600 °C, 60 min; (**d**) 580 °C, 30 min; (**e**) 580 °C, 45 min and (**f**) 580 °C, 75 min.

It has been widely reported that annealing conditions significantly affect the properties of CZTS films. To optimize the annealing conditions for Cu_2_Mg*_x_*Zn_1−*x*_SnS_4_ films, they were annealed under different conditions. Figure 1a–f show the SEM images of the Cu_2_Mg*_x_*Zn_1−*x*_SnS_4_ (*x* = 0.2) films annealed at different conditions. Samples A1, A2 and A3 were annealed for 60 min under a sulfur atmosphere at 540, 580 and 600 °C, respectively. Figure 1a–c shows the SEM images of samples A1, A2 and A3, respectively. As shown in the surface SEM image in Figure 1a, sample A1 contained small nanoparticles (30–100 nm); in addition, a small hole was observed on the surface of the film. Figure 1b shows the SEM image of sample A2; as observed, with an increase in the annealing temperature, the crystalline quality of the Cu_2_Mg*_x_*Zn_1−*x*_SnS_4_ film significantly improved; grain size increased up to 1.4 µm; and the surface became smooth, dense and crack free. However, with a further increase in the annealing temperature to 600 °C, the grain size decreased to 500–900 nm, and more voids and nanoparticles were observed on the surface, as shown in Figure 1c. As shown in Figure 1a–c, sample A2 exhibited optimal crystalline quality, indicating that the optimum annealing temperature was 580 °C. Samples B1, B2 and B3 were annealed at 580 °C under a sulfur atmosphere for 30, 45 and 75 min respectively. Figure 1d–f show the SEM images of samples B1, B2 and B3. Compared to that of sample A2 annealed at 580 °C for 60 min, the crystalline quality of samples B1, B2 and B3 was inferior. Moreover, the surfaces of B1, B2 and B3 were uneven and porous, as shown in Figure 1d–f. As shown in Figure 1b,d,e, with an increase in the annealing time from 30 min and 45 min, the grain size increased from 70–200 nm to 100–500 nm, and then, with a further increase in the annealing time to 60 min, the grain size increased to 100–1400 nm. However, as the film was annealed for a longer time (75 min), the grain size of the Cu_2_Mg*_x_*Zn_1−*x*_SnS_4_ film decreased to 200–700 nm. The surface morphological examination indicated that the Cu_2_Mg*_x_*Zn_1−*x*_SnS_4_ grain growth gradually occurred, and an optimal crystallization quality was achieved by annealing at 580 °C for 60 min.

Table 1 lists the electrical transport parameters of the Cu_2_Mg*_x_*Zn_1−*x*_SnS_4_ (*x* = 0.2) film annealed at different annealing conditions. As observed, the film invariably exhibited *p*-type conductivity. With an increase in the annealing temperature from 540 °C to 600 °C, the carrier concentration first sharply increased from 4.12 × 10^15^ cm^−3^ (sample A1) to 3.29 × 10^18^ cm^−3^ (sample A2), and then decreased to 3.79 × 10^17^ cm^−3^ (sample A3); notably, the resistivity decreased from 9.43 × 10^0^ Ωcm to 1.16 × 10^−1^ Ωcm, and then increased to 1.53 × 10^0^ Ωcm. The mobility decreased from 3.70 × 10^0^ cm^2^ V^−1^ S^−1^ to 1.01 × 10^−1^ cm^2^ V^−1^ S^−1^ and then increased to 7.87 × 10^−1^ cm^2^ V^−1^ S^−1^. Similarly, with an increase in the annealing time from 30 min to 75 min, the carrier concentration first increased from 3.21 × 10^14^ cm^−3^ (sample B1) to 3.79 × 10^15^ cm^−3^ (sample B2), reached the maximum value of 3.29 × 10^18^ cm^−3^ (sample A2) and finally reduced to 4.62 × 10^16^ cm^−3^ (sample B3). Simultaneously, the mobility decreased from 6.02 × 10^0^ cm^2^ V^−1^ S^−1^ for sample B1 to 2.02 × 10^0^ cm^2^ V^−1^ S^−1^ for sample B2, and then reached the minimum 1.01 × 10^−1^ cm^2^ V^−1^ S^−1^ for A2 and finally slightly elevated to 9.32 × 10^−1^ cm^2^ V^−1^ S^−1^ for sample B3. According to the SEM and Hall results, the carrier concentration gradually increases with the annealing time changes from 30 to 60 min, but it starts to decrease when the annealing time increases from 60 to 75 min. It can be explained that when the annealing time increases from 30 to 60 min, the crystallinity of Cu_2_Mg*_x_*Zn_1−*x*_SnS_4_ films is improved, the defects at the grain boundaries are passivated, resulting in the increase of carrier concentration. When the annealing time increases from 60 to 75 min, the crystallization quality is slightly deteriorated, as shown in the previous SEM results, therefore, the carrier concentration decrease. The change of mobility is opposite to that of the carrier concentration. When the annealing time changes from 30 to 60 min, the mobility decreases with the increasing of the carrier concentration, and when the annealing time increases from 60 to 75 min, the mobility starts to increase with the decreasing of the carrier concentration. Finally, it was found when the film was annealed at 580 °C for 60 min, the Cu_2_Mg*_x_*Zn_1–*x*_SnS_4_ film has the optimum crystallization quality and the best electrical performance with the carrier concentration of 3.29 × 10^18^ cm^−3^ and the mobility of 1.01 × 10^−1^ cm^2^ V^−1^ S^−1^. It is concluded that the change of the electrical properties may have great relevance to the defects passivated in the grain boundaries by improving the crystallinity properties.

**Figure 2:**
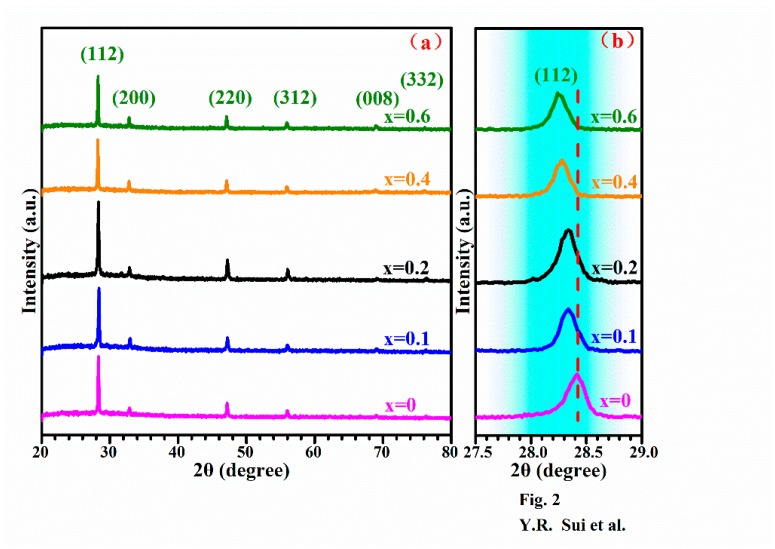
(**a**) XRD spectra of Cu_2_Mg*_x_*Zn_1−*x*_SnS_4_ (0 ≤ *x* ≤ 0.6) thin films. (**b**) Enlarged view of the corresponding (112) diffraction peaks of the Cu_2_Mg*_x_*Zn_1−*x*_SnS_4_ (0 ≤ *x* ≤ 0.6) thin films.

To evaluate the crystalline quality and investigate the existence of impurity phases, the films were subjected to XRD analysis. Figure 2 illustrates the XRD patterns of the Cu_2_Mg*_x_*Zn_1−*x*_SnS_4_ (0 ≤ *x* ≤ 0.6) thin films. As shown in Figure 2a, strong diffraction peaks at 2θ = 28.53, 32.99, 47.33 and 56.17° were observed for all films, which were assigned to the (112), (200), (220) and (312) diffraction planes of kesterite CZTS (JCPDS card no. 26-0575) [26,27]. In addition, two weak peaks were observed at 2θ = 69.27° and 76.44°, which were ascribed to the (008) and (332) planes of kesterite CZTS [28], suggesting that the crystalline quality of the Cu_2_Mg*_x_*Zn_1−*x*_SnS_4_ films was satisfactory. Apart from the diffraction peaks of CZTS, no secondary phase peaks were detected, indicating that Mg doping did not affect the crystal structure of the CZTS film. As observed, with an increase in *x* from 0 to 0.1, the intensity of the (112) peak slightly increased, and then with a further increase in *x* to 0.2, the peak intensity reached the maximum, implying that the crystalline quality of the Cu_2_Mg*_x_*Zn_1−*x*_SnS_4_ thin film with *x* = 0.2 is the best. However, with increasing *x* from 0.2 to 0.6, the intensity of the (112) peak gradually decreased and became the lowest at *x* = 0.6; this gradual deterioration in the crystallinity of the Cu_2_Mg*_x_*Zn_1−*x*_SnS_4_ thin films with increasing *x* was attributed to excessive Mg doping. Figure 2b shows the enlarged view of the (112) peaks. As observed, with an increasing Mg content, the (112) peak unidirectionally shifted to smaller 2θ values, suggesting an increase in the lattice constant of Cu_2_Mg*_x_*Zn_1−*x*_SnS_4_. It is well known that the change of the ion radius in CZTS usually results in the change of lattice parameters [29,30,31]. The occupation of Zn^2+^ sites in the CZTS host lattice by Mg^2+^ ions results in an increase in the Cu_2_Mg*_x_*Zn_1−*x*_SnS_4_ lattice parameters, because the covalent radius of Mg^2+^ (1.36 Å) is larger than that of Zn^2+^ (1.25 Å). Thus, the XRD results indicated that with Mg doping, the phase structure of CZTS did not change, and the Zn^2+^ sites in the CZTS host lattice were occupied by Mg^2+^.

The formation of pure kesterite Cu_2_Mg*_x_*Zn_1−*x*_SnS_4_ cannot be properly confirmed by XRD, because the lattice parameters of CZTS and some possible impurity phases such as tetragonal Cu_2_SnS_3_, cubic ZnS and Cu*_x_*S are similar [32,33]. Therefore, to confirm the formation of pure kesterite Cu_2_Mg*_x_*Zn_1−*x*_SnS_4_, the samples were subjected to Raman spectroscopy analysis.

**Figure 3:**
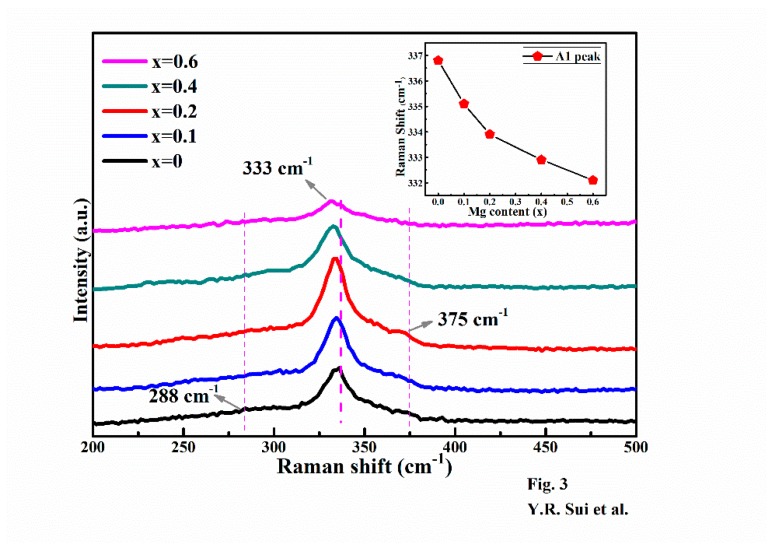
Raman spectra of Cu_2_Mg*_x_*Zn_1−*x*_SnS_4_ (0 ≤ *x* ≤ 0.6) thin films. Inset: The main Raman peaks of A_1_ mode as a function of the Mg content.

Figure 3 shows the Raman spectra of Cu_2_Mg*_x_*Zn_1−*x*_SnS_4_ (0 ≤ *x* ≤ 0.6) films. As shown, the spectra contained the dominant characteristic peak at 333 cm^−1^ and two relatively weak peaks at 288 cm^−1^ and 375 cm^−1^. These Raman peaks were attributed to the A1, A2 and E vibration modes of the S atom in kesterite CZTS, respectively; these results agreed well with those previously reported [34,35]. Notably, no other ternary or binary phase (Cu_2_SnS_3_, SnS_2_, SnS, ZnS) peaks were observed in the Raman spectra. In addition, as shown in Figure 3, with an increase in *x* from 0 to 0.6, the Raman peak, particularly for the peak of A_1_ vibration mode, was slightly red shift systematically. Figure 3 displays the A1 mode peak position variation as a function of the Mg content; as observed, the peak shifted from 336.79 cm^−1^ to 332.13 cm^−1^ with an increase in the Mg content. Combining with the XRD results, the change in the A1 peak position could be ascribed to lattice expansion due to the substitution of the smaller Zn ions by the larger Mg ions in Cu_2_Mg*_x_*Zn_1−*x*_SnS_4_. The redshift in the lattice vibrations were attributed to the lower bonding force of Mg–S than that of Zn–S, resulting from the larger covalent radius of Mg than that of Zn. A similar Raman peak shift caused by ion replacement has been reported in previous studies [36]. Combined with XRD results to analyze the result of Raman spectra, it was found that no other impurity compounds were detected in Cu_2_Mg*_x_*Zn_1−*x*_SnS_4_ films when the *x* was in the range of 0 to 0.6. The pure kesterite Cu_2_Mg*_x_*Zn_1−*x*_SnS_4_ thin films were successfully prepared.

**Figure 4:**
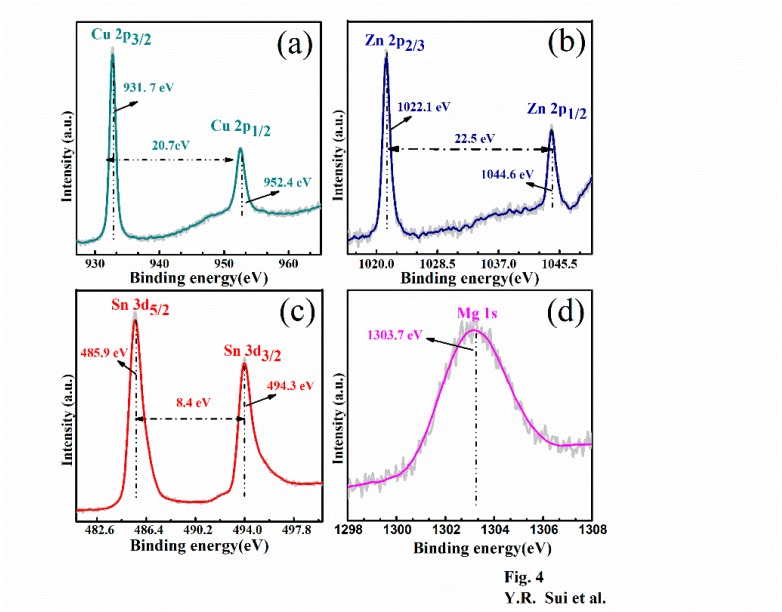
X-ray photoelectron spectroscopy (XPS) spectrum of Cu_2_Mg*_x_*Zn_1−*x*_SnS_4_ (*x* = 0.2) thin films: (**a**) Cu, (**b**) Zn, (**c**) Sn and (**d**) Mg.

Notably, the chemical composition of the Cu_2_Mg*_x_*Zn_1−*x*_SnS_4_ films and the chemical bonding states of the constituents significantly affect the solar cell performance. Hence, the Cu_2_Mg*_x_*Zn_1−*x*_SnS_4_ films were characterized by XPS. Figure 4a–d show the XPS profiles of the constituent metals (Cu, Zn, Sn and Mg) of the representative Cu_2_Mg*_x_*Zn_1−*x*_SnS_4_ (*x* = 0.2) sample. Figure 4a displays the Cu 2p XPS profile. The two peaks at 952.4 eV and 931.7 eV were attributed to Cu 2p1/2 and Cu 2p3/2. In addition, the peak separation value agreed well with the standard value of 20.7 eV, indicating that Cu was present in the +1 combined-state [37]. Figure 4b illustrates the XPS spectrum of Zn 2p. The two peaks located at 1044.6 eV and 1022.1 eV were attributed to Zn 2p1/2 and Zn 2p3/2, respectively, the splitting energy was 22.5 eV. The splitting value is consistent with the standard value of 22.97 eV, which confirms that Zn exists in a +1 state [38]. The Sn 3d XPS profile is displayed in Figure 4c. As observed, two peaks of Sn 3d_3/2_ and Sn 3d_5/2_, situated at 494.3 and 485.9 eV were detected; the peak separation value was 8.4 eV, which agreed with the standard value, implying that Sn was in the Sn^4+^ oxidation state [39]. Figure 4d presents the Mg 1s XPS profile; the peak at 1303.7 eV was assigned to the Mg 1s core level, indicating the presence of divalent Mg^2+^ [24]. According to the results of XPS, the valence states of Cu, Zn, Mg and Sn were +1, +2, +4 and +2 respectively. This further confirmed the substitution of Zn in CZTS by Mg, agreeing well with the XRD and Raman results.

**Figure 5:**
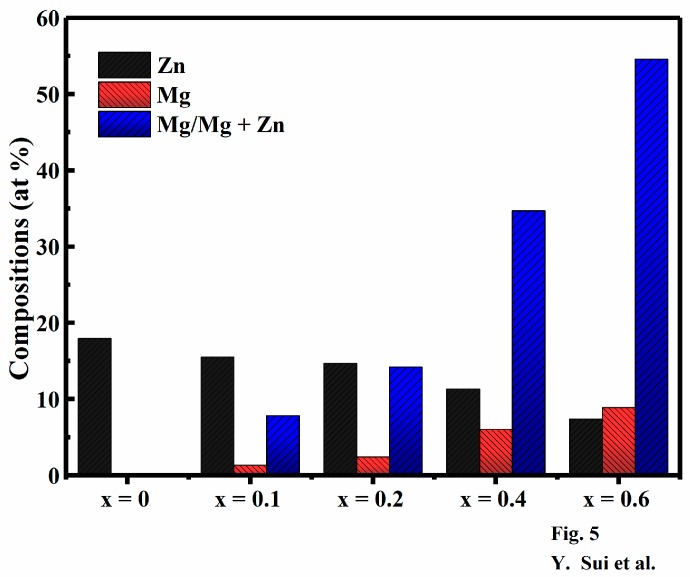
Energy dispersive X-ray spectroscopy (EDS) composition analyses of Cu_2_Mg*_x_*Zn_1−*x*_SnS_4_ (0 ≤ *x* ≤ 0.6) thin films.

The atomic contents of Cu, Zn, Sn, S and Mg in the Cu_2_Mg*_x_*Zn_1−*x*_SnS_4_ (0 ≤ *x* ≤ 0.6) films are listed in Table 2. When the percentages of Mg/(Mg + Zn) for the precursor solution of Cu_2_Mg*_x_*Zn_1−*x*_SnS_4_ (0 ≤ *x* ≤ 0.6) were 0, 10, 20, 40 and 60, the percentages of Mg/(Mg + Zn) in Cu_2_Mg*_x_*Zn_1−*x*_SnS_4_ films were 0, 7.79, 14.22, 34.72 and 54.61, respectively. Notably, the elemental loss during annealing and the preparation process cannot be neglected; nonetheless, the Mg/(Mg + Zn) ratio in the Cu_2_Mg*_x_*Zn_1−*x*_SnS_4_ films increased with an increase in the Mg content in the precursor solution. Figure 5 summarizes the atomic contents of the constituent elements of the Cu_2_Mg*_x_*Zn_1−*x*_SnS_4_ (0 ≤ *x* ≤ 0.6) films, according to the energy dispersive X-ray spectroscopy (EDS) results presented in Table 2. As observed, the atomic content of Mg gradually increased with a decrease in the atomic content of Zn from 17.95 to 7.39; moreover, the Mg/(Mg + Zn) ratio also increased. This indicated that Mg was incorporated into the CZTS lattice, replacing Zn. Furthermore, the changes in the atomic contents of other elements in the Cu_2_Mg*_x_*Zn_1−*x*_SnS_4_ films were negligible. The result is in good agreement with the conclusion that Mg will substitute the site of Zn obtained from the analysis result of XRD and Raman.

**Figure 6:**
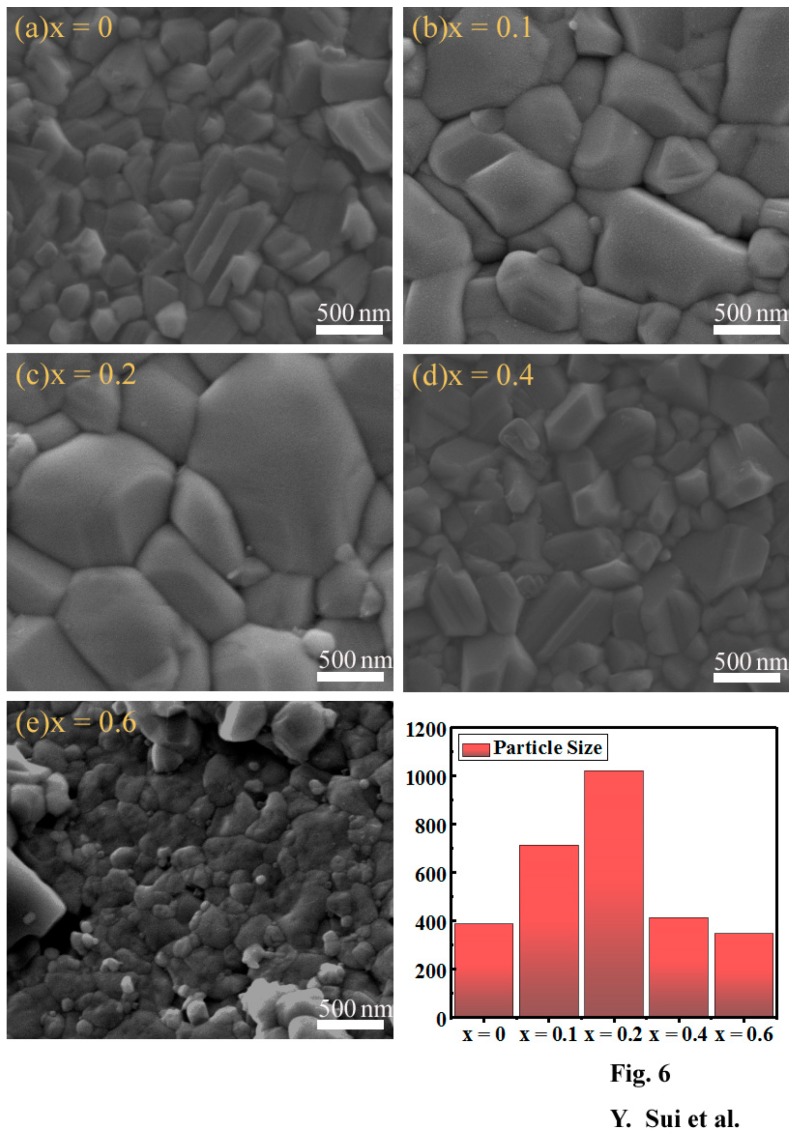
SEM images of Cu_2_Mg*_x_*Zn_1−*x*_SnS_4_ (0 ≤ *x* ≤ 0.6) thin films with different Mg content: (**a**) *x* = 0, (**b**) *x* = 0.1, (**c**) *x* = 0.2, (**d**) *x* = 0.4 and (**e**) *x* = 0.6. Inset: The average diameter of particles as a function of the Mg content.

To determine the effect of the Mg content on the crystalline quality of the Cu_2_Mg*_x_*Zn_1−*x*_SnS_4_ (0 ≤ *x* ≤ 0.6) films, the films were detected by SEM as shown in Figure 6a–e. Figure 6a displays the surface SEM images of the Cu_2_Mg*_x_*Zn_1−*x*_SnS_4_ film with *x* = 0. As observed, the film consisted of irregular nanoscale grains (40–500 nm). Moreover, the surface of the film was relatively rough, but compact. Obviously, the irregular grain boundaries and small particles are not conducive to the improvement of the efficiency for the CZTS solar cells. As shown in Figure 6b, with an increase in *x* to 0.1, the film crystallinity enhanced and the grain size increased to 400–1200 nm. Furthermore, the surface morphology was significantly improved and become smooth and compact. With a further increase in the value of *x* to 0.2, the film surface became very flat and dense, as displayed in Figure 6c; in addition, the grain size further increased to 0.7–1.5 µm, which was conducive to achieving high efficiencies for CZTS solar cells. Figure 6d shows the SEM image of the Cu_2_Mg*_x_*Zn_1−*x*_SnS_4_ film with *x* = 0.4. As observed, the grain size sharply decreased to 300–900 nm, but the grains were larger than those of Cu_2_Mg*_x_*Zn_1−*x*_SnS_4_ with *x* = 0, and densely stacked. The crystalline quality of the Cu_2_Mg*_x_*Zn_1−*x*_SnS_4_ film continued to deteriorate with further increase in *x* to 0.6. As shown in Figure 6e, the grain size of Cu_2_Mg*_x_*Zn_1−*x*_SnS_4_ film reduced to 200–400 nm, occasionally, a few larger grains were observed on the film surface. As seen, the surface morphology of the film with *x* = 0.6 was uneven and irregular. The bar chart in Figure 6 shows the average particle diameter as a function of the Mg content. As seen, the average size gradually increased with an increase in the value of *x* from 0 to 0.2 and reached the maximum at *x* = 0.2, then with further increase in *x* from 0.2 to 0.6, the size sharply decreased. It is well known that the good grain growth and smooth surface is of great significance to the fabrication of high power conversion efficiency (PCE) CZTS solar cells. Because the absorption layer with larger particle size can reduce the grain boundaries area, which is conducive to decrease the recombination of photon-generated carrier and increase the efficiency of CZTS solar cells. Based on the results of SEM, when the value of *x* was 0.2, it was concluded that the crystallization quality of Cu_2_Mg*_x_*Zn_1−*x*_SnS_4_ films achieved the best results, the grain size was the largest and the surface was the smoothest and denser, which is most suitable for the absorber layer of the CZTS solar cells.

**Figure 7:**
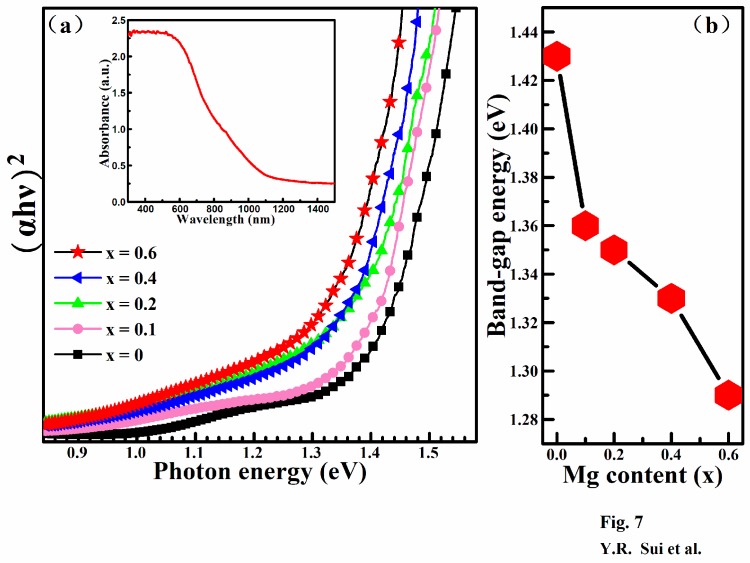
(**a**) The plot of (ahυ)^2^ vs hυ for the absorption spectra. The inset shows the UV–vis absorption spectra of the representative Cu_2_Mg*_x_*Zn_1−*x*_SnS_4_ with *x* = 0.2; (**b**) Band gap variation as a function of the Mg content.

UV-Vis-NIR spectroscopy was carried out to investigate the influence of Mg content on the band gap (E_g_) values of the Cu_2_Mg*_x_*Zn_1−*x*_SnS_4_ (0 ≤ *x* ≤ 0.6) thin films. Figure 7a illustrates the plots of (αhυ)^2^ against hυ for the films, where α and hυ are the absorption coefficient and photon energy, respectively. The E_g_ values for the Cu_2_Mg*_x_*Zn_1−*x*_SnS_4_ (0 ≤ *x* ≤ 0.6) films can be obtained by optical absorption measurements, according to Tauc’s relation [40]:

(αhυ) = A(hυ − E_g_)^n^, (1)

where A is a constant, n = 1/2, 3/2, 2 and 3 for the allowed direct, forbidden direct, allowed indirect and forbidden indirect transitions, respectively [41]. In general, Cu_2_Mg*_x_*Zn_1−*x*_SnS_4_ is regarded as a direct band gap semiconductor, therefore, n = 1/2. The values of E_g_ for the Cu_2_Mg*_x_*Zn_1−*x*_SnS_4_ thin films with *x* = 0, 0.1, 0.2, 0.4 and 0.6 calculated according to Tauc’s relation were 1.43, 1.36, 1.35, 1.33 and 1.29 eV, respectively. The inset of Figure 7a shows the UV–vis absorption spectra of the representative Cu_2_Mg*_x_*Zn_1−*x*_SnS_4_ with *x* = 0.2. It was found that the Cu_2_Mg*_x_*Zn_1−*x*_SnS_4_ film had a stronger absorption intensity in the short wavelength range, which is suitable as the absorber layer of the CZTS solar cells. Figure 7b shows the variation in the band gap energy as a function of the Mg content. As observed, the E_g_ value reduced from 1.43 to 1.29 eV with an increase in *x* from 0 to 0.6, which can be ascribed to the change in the lattice parameter, resulting from the occupation of Zn sites by Mg. According to the first principles calculation results for the CZTS semiconductor, the minimum of the conduction band depends on the Sn 3d and S 3p antibonding orbitals and the maximum of the valence band is primarily related to *p*–*d* hybridization between Cu and S [42,43,44]. In the present work, the Mg element will take the site of Zn in CZTS, which will not affect the band gap of CZTS based on the theoretical analysis mentioned above. However, the band gap of Cu_2_Mg*_x_*Zn_1−*x*_SnS_4_ linearly varied as *x* increased from 0 to 0.6. Similar phenomena that the band gap of CZTS changes regularly because Zn is replaced by other elements (Cd, Ge) have been mentioned in previous studies [33,44], they ascribed the change to an increase in the unit cell volume, which led to a reduction in the antibonding component of the *s–p* and *s–s* hybridization between S^2−^ and Sn^4+^, resulting in a decrease in the minimum of the conduction band. In the present work, the substitution of Zn by Mg increased the volume of the unit cell and reduced the antibonding component of *s–p* and *s–s* hybridization between S^2−^ and Sn^4+^. Hence, the minimum of the conduction band and the band gap of Cu_2_Mg*_x_*Zn_1−*x*_SnS_4_ gradually decreased with increasing Mg content.

The conductivity (ρ), carrier concentration (n) and mobility (μ) for the Cu_2_Mg*_x_*Zn_1−*x*_SnS_4_ (0 ≤ *x* ≤ 0.6) thin films were determined by the van der Pauw method at room temperature and the results are presented in Table 3. Tests were repeated on the same sample to ensure precision and reliability of the electrical performances of the Cu_2_Mg*_x_*Zn_1−*x*_SnS_4_ films. All samples with different Mg contents exhibited *p*-type semiconductor characteristics. In addition, as the value of *x* increased from 0 to 0.2, the carrier concentration of the Cu_2_Mg*_x_*Zn_1−*x*_SnS_4_ films increased from 6.95 × 10^16^ cm^−3^ to 3.29 × 10^18^ cm^−3^. However, with a further increase in *x* from 0.2 to 0.6, the carrier concentration gradually decreased to 2.02 × 10^17^ cm^−3^. Simultaneously, the mobility decreased from 2.63 × 10^0^ cm^2^ V^−1^ S^−1^ to 1.01 × 10^−1^ cm^2^ V^−1^ S^−1^ with an increase in *x* from 0 to 0.2, and then, the mobility increased to 1.43 × 10^0^ cm^2^ V^−1^ S^−1^ as *x* increased to 0.6. The increase in the carrier concentration with an increase in *x* from 0 to 0.2 was attributed to the passivation of grain boundary defects, resulting from an improvement in the crystalline quality of the Cu_2_Mg*_x_*Zn_1−*x*_SnS_4_ thin film. As previously reported for K-doped and Na-doped CZTSSe and CIGS solar cells [45,46], the carrier concentration markedly improved because of passivation of grain boundary defects, resulting from an improvement in the crystallization properties. Moreover, the decrease in the carrier concentration observed in this study with an increase in *x* from 0.2 to 0.6 was attributed to the deterioration of crystalline quality due to the small grain size, multi-hole and irregular surface morphology. Notably, the Cu_2_Mg*_x_*Zn_1−*x*_SnS_4_ film with *x* = 0.2 exhibited an optimal electrical conductivity.

## 4. Conclusions

In conclusion, we have prepared Cu_2_Mg*_x_*Zn_1−*x*_SnS_4_ films with different Mg contents and investigated the influence of the annealing temperature and time on the performance of the films. The optimal annealing temperature and time was found to be 580 °C and 60 min, respectively. Moreover, under the optimal annealing conditions, we investigated the effect of Mg content on the performance of the Cu_2_Mg*_x_*Zn_1−*x*_SnS_4_ films in detail. It was found that the Cu_2_Mg*_x_*Zn_1−*x*_SnS_4_ films were ideal for use as absorption layers in solar cells because of their continuous tunable band gaps, favorable photoelectric performance and high crystallinity. With an increase in *x* from 0 to 0.6, the band gap increased from 1.43 to 1.29 eV. Notably, the continuous tunable band gap could facilitate the tuning of band alignment at the Cu_2_Mg*_x_*Zn_1−*x*_SnS_4_/CdS heterojunction by changing the Mg content. Furthermore, the Cu_2_Mg*_x_*Zn_1−*x*_SnS_4_ film with *x* = 0.2 exhibited superior crystallinity and surface morphology compared to other Cu_2_Mg*_x_*Zn_1−*x*_SnS_4_ films. Meanwhile, at *x* = 0.2, the electrical conductivity of Cu_2_Mg*_x_*Zn_1−*x*_SnS_4_ film reached the optimal level, with a carrier concentration of 3.29 × 10^18^ cm^−3^ and a mobility of 1.01 × 10^−1^ cm^2^ V^−1^ S^−1^. Notably, after being annealed at 580 °C for 60 min, the Cu_2_Mg*_x_*Zn_1−*x*_SnS_4_ film with the optimal Mg/(Mg + Zn) ratio of 0.2 exhibited favorable photoelectric performance and enhanced crystalline quality, making it a promising candidate for the preparation of high-efficiency solar cells with tunable band gap absorption layers.

## Figures and Tables

**Table 1:** Electrical properties of the Cu_2_Mg*_x_*Zn_1−*x*_SnS_4_ (*x* = 0.2) thin films annealed at different annealing conditions.

Sample	ρ (Ω.cm)	n (cm^−3^)	μ (cm^2^ V^−1^ S^−1^)	Type
**A1**	9.43 × 10^0^	4.12 × 10^15^	3.70 × 10^0^	*p*
**A2**	1.16 × 10^−1^	3.29 × 10^18^	1.01 × 10^−1^	*p*
**A3**	1.53 × 10^0^	3.79 × 10^17^	7.87 × 10^−1^	*p*
**B1**	7.99 × 10^1^	3.21 × 10^14^	6.02 × 10^0^	*p*
**B2**	8.97 × 10^0^	3.79 × 10^15^	2.02 × 10^0^	*P*
**B3**	4.53 × 10^0^	4.62 × 10^16^	9.32 × 10^−1^	*P*

**Table 2:** EDS composition analyses of the Cu_2_Mg*_x_*Zn_1−*x*_SnS_4_ (0 ≤ *x* ≤ 0.6) thin films.

Sample	Cu (at %)	Zn (at %)	Mg (at %)	Sn (at %)	S (at %)	Mg/(Mg + Zn) (at %)
***x* = 0**	25.07	17.95	0	10.30	46.98	0
***x* = 0.1**	25.34	15.51	1.31	10.03	47.81	7.79
***x* = 0.2**	25.52	14.66	2.43	10.61	46.78	14.22
***x* = 0.4**	25.31	11.32	6.02	10.32	47.03	34.72
***x* = 0.6**	25.08	7.39	8.89	10.77	47.87	54.61

**Table 3:** Electrical properties of the Cu_2_Mg*_x_*Zn_1−*x*_SnS_4_ (0 ≤ *x* ≤ 0.6) thin films.

Sample	ρ (Ω.cm)	n (cm^−3^)	μ (cm^2^ V^−1^ S^−1^)	Type
***x* = 0**	3.73 × 10^1^	6.95 × 10^16^	2.63 × 10^0^	*p*
***x* = 0.1**	3.21 × 10^1^	2.46 × 10^17^	3.23 × 10^−1^	*p*
***x* = 0.2**	1.16 × 10^−1^	3.29 × 10^18^	1.01 × 10^−1^	*p*
***x* = 0.4**	1.92 × 10^−1^	1.21 × 10^18^	1.22 × 10^0^	*p*
***x* = 0.6**	1.12 × 10^0^	2.02 × 10^17^	1.43 × 10^0^	*p*
